# Effects of Echinocandins in Combination with Nikkomycin Z against Invasive Candida albicans Bloodstream Isolates and the *fks* Mutants

**DOI:** 10.1128/AAC.00619-17

**Published:** 2017-10-24

**Authors:** Yuk-Yam Cheung, Mamie Hui

**Affiliations:** Department of Microbiology, Faculty of Medicine, The Chinese University of Hong Kong, Prince of Wales Hospital, Shatin, New Territories, Hong Kong

**Keywords:** Candida albicans, FKS, echinocandin, nikkomycin Z

## Abstract

We evaluated the *in vitro* and *in vivo* effects of nikkomycin Z combined with an echinocandin (anidulafungin or micafungin) against two Candida albicans isolates and their lab-derived echinocandin-resistant *fks* mutants with FKS1 S645Y and FKS1 S645P. Synergistic effects were observed in all tested strains (fractional inhibitory concentration index, <0.5). Enhanced survival was observed in an immunocompromised murine model (log-rank test, *P* < 0.02). Our study demonstrated the therapeutic potential of nikkomycin Z-echinocandin combinations in managing echinocandin resistance.

## TEXT

Echinocandins are considered first-line treatment for invasive Candida infections (1). However, treatment failures associated with resistant isolates harboring *fks* hot-spot mutations have been reported (2, 3). Nikkomycin Z is a chitin synthase inhibitor with potential therapeutic effects against Candida infections (4). Moreover, *in vitro* synergistic effects were reported when nikkomycin Z was combined with echinocandins against Candida isolates (5, 6). However, the effects of the combination *in vivo* are not yet available. In this study, we evaluated the *in vitro* effects of nikkomycin Z combined with an echinocandin (anidulafungin or micafungin) against two Candida albicans isolates (ATCC 90028 and blood culture isolate CA 46503) and their lab-derived echinocandin-resistant *fks* mutants. The *in vivo* effects of the antifungal combinations were studied in an immunosuppressed murine model.

Anidulafungin (Pfizer, Inc., USA), micafungin (Astellas Pharma, Inc., Japan), and nikkomycin Z (Sigma, USA) were used throughout the study. Spontaneous *fks* mutants of the two C. albicans parent strains were isolated by plating 10 μl (∼10^8^ cells) Sabouraud broth culture onto Sabouraud dextrose agar plates containing 8 μg/ml micafungin. Resistant isolates were reinoculated onto fresh plates containing 8 μg/ml micafungin to confirm the nonsusceptible phenotype. The isolates were characterized by *fks* hot-spot sequencing and antifungal susceptibility tests according to the CLSI broth microdilution method (7, 8). The nikkomycin Z MIC was the lowest drug concentration exhibiting 50% reduction in turbidity after 24 h of incubation. *In vitro* drug interactions were assessed by checkerboard assays with the fractional inhibitory concentration index (FICI) interpreted as follows: ≤0.5, synergistic; 0.5 to ≤4, indifferent; and >4, antagonistic (9). Tests were done in duplicate. C. albicans *fks1* hot spot 1 was amplified with the forward primer BIO-1HS1F 5′-biotin-AATGGGCCGGTGCTCAACA-3′ and reverse (also sequencing) primer 1HS1-seq 5′-TTCACCATTACATCTCAT-3′. Corresponding primers for *fks1* hot spot 2 were BIO-1HS2F 5′-biotin-AAGATTGGTGCTGGTATGGG-3′ and 1HS2-seq 5′-ACCTCTTTCAATCAATTCTTGAACAAC-3′ (10). The *fks* hot spots were examined by pyrosequencing (PyroMark Q24; Qiagen, CA).

Murine models of systemic candidiasis were established in ICR mice (weighing ∼20 g) by intravenous inoculation of 100 μl (in a 1-ml syringe; Terumo, USA) of the four C. albicans strains (2 parents and 2 *fks* mutants; 5 × 10^6^ yeast cells) via tail vein (11). The mice were immunosuppressed by intraperitoneal injection of 100 mg/kg dexamethasone on days −3, 0, 7, and 14. Therapy began 1 day postinfection and continued for 12 days. A dose of 5 mg/kg of and echinocandin (anidulafungin or micafungin) was given subcutaneously once daily (12, 13). A dose of 10 mg/kg of nikkomycin Z was given subcutaneously twice daily (14). All mice were held for 17 days and monitored daily for mortalities. There were 10 mice per group. Kaplan-Meier survival plots were analyzed by a log-rank test (Prism, version 7.03; GraphPad Software, CA). *P* values were considered significant at the 0.05 level. All animal studies were approved by the Institutional Review Board.

**FIG 1:**
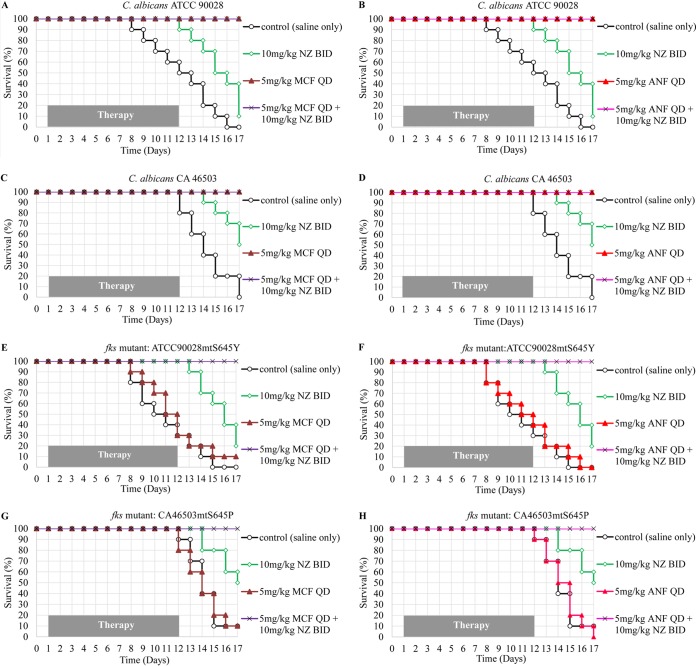
Survival curves of the immunosuppressed mice infected with C. albicans parent strains (ATCC 90028 and CA 46503) and their lab-derived *fks* mutants (ATCC90028mtS645Y and CA46503mtS645P). NZ, nikkomycin Z; MCF, micafungin; ANF, anidulafungin; QD, once daily; BID, twice daily.

Two spontaneous *fks* mutants, ATCC90028fksmtS645Y and CA46503fksmtS645P, were derived from C. albicans ATCC 90028 and CA 46503, respectively. Both mutants harbored a single substitution mutation in the *fks1* hot-spot region, and both were homozygous. The MIC results and FICIs are shown in Table 1. The *fks* mutants showed 32-fold elevations in MIC for anidulafungin and micafungin. Synergistic effects (nikkomycin Z and echinocandin) were observed in the parent strains and the *fks* mutants. Kaplan-Meier survival curves are shown in Fig. 1. In the saline treatment control group, C. albicans ATCC 90028 was more virulent than CA 46503. The killing rates of the parent strains and their derived *fks* mutants were similar. Monotherapy with nikkomycin Z prolonged the survival of all infected mice (log-rank test, *P* < 0.01), but the survival rates declined once the nikkomycin Z was discontinued. Treatment with either anidulafungin or micafungin improved the survival of mice infected with the parent strain but not in those infected with the *fks* mutants. Combination treatment with nikkomycin Z and either echinocandin significantly improved the survival rate of mice infected with the *fks* mutants compared with that of mice treated with nikkomycin Z or echinocandin monotherapy (log-rank test, *P* < 0.02).

**TABLE 1:** MIC and FICI values of C. albicans parent strains and their lab-derived *fks* mutants

C. albicans strain	FKS hot-spot region	MIC (μg/ml) of^*a*^:			Drug combination^*b*^	MIC of combination (μg/ml)	FICI^*c*^	Interpretation
		ANF	MCF	NZ				
ATCC 90028	Wild type	0.03 (S)	0.03 (S)	4	ANF + NZ	0.004 + 1	0.38	Synergy
					MCF + NZ	0.004 + 1	0.38	Synergy
ATCC 90028mtS645Y	FKS1 S645Y	1 (R)	1 (R)	4	ANF + NZ	0.125 + 1	0.38	Synergy
					MCF + NZ	0.125 + 1	0.38	Synergy
CA 46503	Wild type	0.03 (S)	0.03 (S)	4	ANF + NZ	0.004 + 1	0.38	Synergy
					MCF + NZ	0.004 + 1	0.38	Synergy
CA 46503mtS645P	FKS1 S645P	1 (R)	1 (R)	4	ANF + NZ	0.125 + 1	0.38	Synergy
					MCF + NZ	0.125 + 1	0.38	Synergy

a S, sensitive; R, resistant.

b ANF, anidulafungin; MCF, micafungin; NZ, nikkomycin Z.

c FICI, fractional inhibitory concentration index.

In this study, spontaneous C. albicans fks mutants were derived to assess the effects of combinations of nikkomycin Z and echinocandins. The mutations, *fks1* T1933C (FKS1 S645P) and *fks1* C1934A (FKS1 S645Y), and their associated elevations in echinocandin MIC were also observed previously (15, 16). The maximum plasma concentrations of anidulafungin, micafungin, and nikkomycin Z were reported to be, respectively, 49.5, 53, and 49.5 μg/ml in murine (13, 17, 18) and 8, 16, and 6.42 μg/ml in human adults (19, 20). Our *in vitro* synergistic effects were observed at achievable plasma concentrations in murine and humans, suggesting that the effects are potentially useful *in vivo*. Although the mechanism of the synergy is not fully understood, it was reported that chitin synthesis was upregulated as a result of cell wall salvage pathways when C. albicans isolates were exposed to caspofungin (21). The simultaneous inhibition of chitin synthase and β-1,3-glucan synthase by nikkomycin Z and an echinocandin probably renders the salvage pathway useless and impairs construction of the cell wall.

The *in vivo* response in this study correlated well with the resistance phenotype. Monotherapy with echinocandin did not produce significant survival in *fks* mutant-infected mice. Their survival was enhanced by nikkomycin Z treatment; however, similar to a previous report, survival declined when treatment was discontinued (14). Combination treatment with nikkomycin Z and echinocandin prevented such a decline and significantly improved survival of the *fks* mutant-infected mice.

In contrast to previous reports that used immunocompetent murine models, presence of the *fks* mutations in C. albicans isolates was not associated with decreased virulence in our immunosuppressed murine model (15, 22). The use of dexamethasone as an immunosuppressant may have affected the virulence results. To the best of our knowledge, this is the first report to demonstrate the *in vivo* therapeutic effects of combined nikkomycin Z and echinocandin in treating *fks* mutation-associated echinocandin-resistant C. albicans infections. One limitation of this study was that we evaluated only one dosing regimen (nikkomycin Z at 10 mg/kg twice daily). Future studies are needed to determine the dose-dependent effect of nikkomycin Z.
